# Impact of Early Rhythmic Training on Language Acquisition and Electrophysiological Functioning Underlying Auditory Processing: Feasibility and Preliminary Findings in Typically Developing Infants

**DOI:** 10.3390/brainsci11111546

**Published:** 2021-11-21

**Authors:** Chiara Dondena, Valentina Riva, Massimo Molteni, Gabriella Musacchia, Chiara Cantiani

**Affiliations:** 1 Child Psychopathology Unit, Scientific Institute, IRCCS Eugenio Medea, Bosisio Parini, 23842 Lecco, Italy; chiara.dondena@lanostrafamiglia.it (C.D.); valentina.riva@lanostrafamiglia.it (V.R.); massimo.molteni@lanostrafamiglia.it (M.M.); 2 Department of Audiology, School of Health Sciences, University of the Pacific, Stockon, CA 95211, USA; gmusacchia@pacific.edu

**Keywords:** music training, infants, language acquisition, EEG/ERP, auditory processing

## Abstract

Previous evidence has shown that early auditory processing impacts later linguistic development, and targeted training implemented at early ages can enhance auditory processing skills, with better expected language development outcomes. This study focuses on typically developing infants and aims to test the feasibility and preliminary efficacy of music training based on active synchronization with complex musical rhythms on the linguistic outcomes and electrophysiological functioning underlying auditory processing. Fifteen infants participated in the training (RTr+) and were compared with two groups of infants not attending any structured activities during the same time frame (RTr−, N = 14). At pre- and post-training, expressive and receptive language skills were assessed using standardized tests, and auditory processing skills were characterized through an electrophysiological non-speech multi-feature paradigm. Results reveal that RTr+ infants showed significantly broader improvement in both expressive and receptive pre-language skills. Moreover, at post-training, they presented an electrophysiological pattern characterized by shorter latency of two peaks (N2* and P2), reflecting a neural change detection process: these shifts in latency go beyond those seen due to maturation alone. These results provide preliminary evidence on the efficacy of our training in improving early linguistic competences, and in modifying the neural underpinnings of auditory processing in infants.

## 1. Introduction

Language and music are fundamental human attributes characterized by a high degree of complexity. Surprisingly, the process of acquisition of such complex systems is relatively fast and efficient. The first months of life seem to be particularly crucial for language acquisition: infants approach language during a sensitive, probably critical period, with a set of basic skills including neuropsychological, perceptual, domain-general computational and cognitive functions. In particular, auditory processing, or how we encode, analyze and interpret auditory input, underlies the infant’s ability to construct phonemic maps of their native language and detect the linguistic features available in the acoustic input [1,2]. Music acquisition is thought to proceed similarly, beginning with the auditory processing of frequencies and beats and continuing through enculturation, whereby representations of culture-specific music that infants hear are established [3]. Despite the fact that language and music acquisition appear to proceed similarly and both skills access the same central nervous system structures, little is known about the relationship between the two skills during early development. This is particularly important considering converging evidence suggesting that the integrity of early auditory processing is a robust predictor of oral and written language abilities at later ages [4,5,6,7,8].

It is widely accepted that the ability to process acoustic information is impaired in individuals (children and adults) with developmental language disorder (DLD) and learning disabilities (LD) [9,10]. Importantly, auditory processing is impaired, even in preverbal infants at various degrees of risk for language impairment, including familial risk for DLD and LD [5,11,12]. Results from our own research group agree with these findings. Previous results obtained from the longitudinal cohort recruited at the Medea BabyLab allowed us to identify the electrophysiological markers of auditory processing that (a) differentiate infants/toddlers with vs. without familial risk for DLD and LD [6,13], and infants/toddlers who are at-risk for different clinical conditions [14]; and (b) are robust predictors of later linguistic skills [6,13,15,16].

As already mentioned, neuropsychological, sensory and cognitive deficits involved in language acquisition are not limited to children with clinical manifestations of the disorder, but also in young children and infants at various degrees of risk for language impairment. This suggests that training and interventions administered very early in life may be effective at improving the language acquisition trajectory of these individuals. In fact, it has recently been proposed that targeted training could be implemented at very early ages, ideally in the first years of life, when cerebral plasticity is known to be maximal [17]. For example, interactive acoustic experience has been shown to enhance and accelerate the maturation of auditory processing in typically developing infants, optimizing the cortical representations that will ultimately support later language, with better expected outcome on later language development [18,19].

A promising tool to improve and promote auditory processing very early in life is active music training. It is well known that music and language share many common encoding and cognitive skills, such as temporal processing. At the acoustic level, music and language use pitch, timing and timbre cues to convey information [20]. At the cognitive level, they require similar memory and attention skills, as well as the ability to integrate discrete acoustic events into a coherent perceptual stream according to specific syntactic rules [21]. Due to the degree of overlapping skills used in acquisition, it has been proposed that training one of the two domains might have positive effects on the other [22].

The overlapping skills hypothesis of speech and music acquisition is supported by several sets of data. First, in older children and adults, active music making has been shown to have wide-ranging positive effects on several neuropsychological, sensorial and cognitive processes, such as auditory processing of both non-speech and speech stimuli [22,23,24,25,26], and cognitive skills such as attention, auditory memory, linguistic and reading skills [26,27]. Interestingly, music training has been shown to be successful in remediating these skills, even in populations with (or at risk for) DLD and LD [28,29,30]. Further work in typical populations has shown that musical training is associated with faster and larger brainstem responses [31,32] and that cortical entrainment is enhanced with years of musical training [33]. In addition, it has been reported that the effect of music training is evident not only at the functional level, but also at the structural level [34,35,36,37], since several studies of neural plasticity in experienced musicians have demonstrated modification. Moreover, the fact that these modifications are correlated with the number of years of musical practice suggests a causal link between the extent or duration of musical experience and functional/structural modifications [31,32]. A final set of evidence, particularly important for the framework of this study, suggests that the functional and structural modification induced by music training might be relevant—presumably, extremely relevant—very early in life, as infants and even newborns have demonstrated a specific sensitivity to musical features such as rhythm [38,39].

To date, a few studies have specifically investigated the effect of active music training on infants’ perceptual and cognitive skills, with very promising results on communicative development [40] and on the neural processing of both speech and non-speech sounds [41]. The only attempt to apply a passive music-listening intervention to promote the development of infants at familial risk for dyslexia has been conducted within the “DyslexiaBaby study” [42]. The authors reported previous findings that support the effects of both active [40] and passive [43] music interventions on auditory processing and language skills; in their study, infants were exposed to recorded children’s songs at home in order to assess the effect of passively hearing recorded music. The preliminary results of this study demonstrated that this kind of stimulation was unable to affect auditory electrophysiological responses or pre-communicative development. The authors suggested that the merely passive nature of the exposure to music was probably not enough to modulate the development of these abilities. Despite finding no effect of the intervention, the authors reported correlations between the amount of informal musical activities at home or musical play at school (e.g., dancing or moving to music, drumming or tapping rhythms) and both pre-communicative development scores and the maturity of the electrophysiological responses to auditory stimuli (e.g., larger amplitudes and shorter latencies of several components). These latter results highlight the beneficial role of more active musical activities in facilitating early development.

The goal of the present study is to test the feasibility and efficacy of innovative, ecological early rhythmic training based on an enriched active auditory and musical experience on the linguistic outcomes and electrophysiological functioning underlying auditory processing.

## 2. Materials and Methods

### 2.1. Participants

Parents of 45 infants were asked to participate in this study. Children were recruited at 6 months of age via local advertisements in the area of Lecco, Como and Monza-Brianza (Northern Italy) between April 2017 and October 2019. The study was conducted in accordance with the Declaration of Helsinki, and the protocol was approved by the Eugenio Medea Scientific Institute Ethic and Scientific Committees (Ricerca Corrente “2016”, id. 298; and Ricerca Corrente “2019”, id. 629). Written informed consent was obtained from all parents of children involved in the study prior to testing.

**Figure 1:**
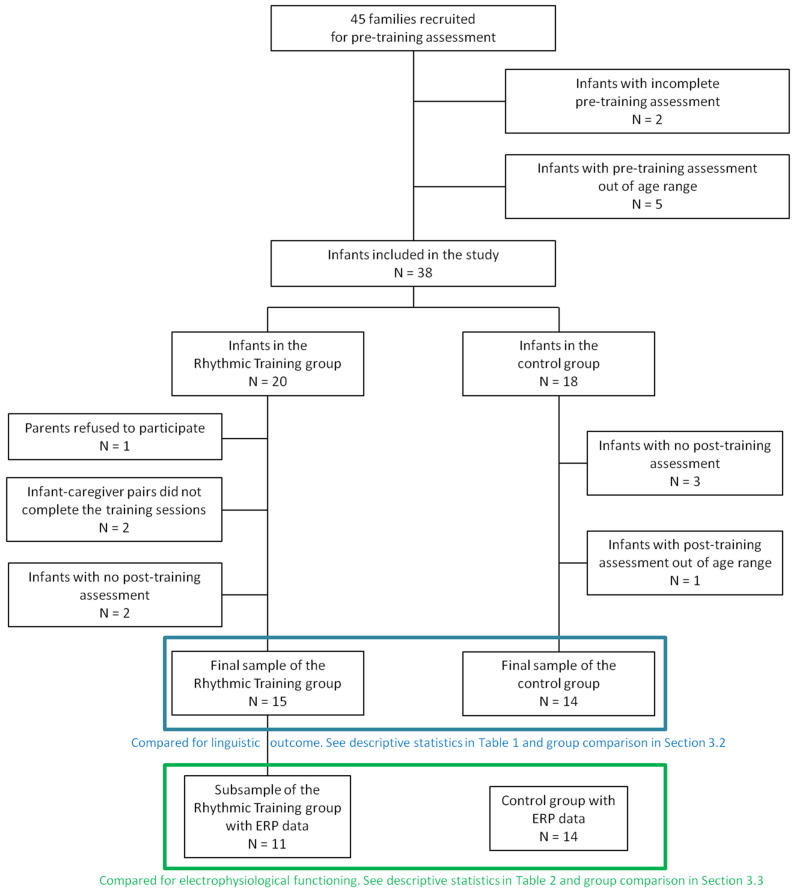
Flow diagram of the families who participated in the study.

Sociodemographics data and prenatal and perinatal information were collected using an ad hoc questionnaire filled by parents, and infants’ cognitive level was assessed during the first visit. Infants were included if (1) both parents were native Italian speakers; (2) gestational age was ≥37 weeks; (3) birthweight was ≥2500 g; (4) Bayley Cognitive Scale Score was ≥7; and (5) first-degree relatives had no certified diagnosis of intellectual deficiency or neurodevelopmental disorders. Two of the 45 children did not complete the pre-training assessment and five completed once they were out of the required age range due to family difficulties in scheduling the visits. Therefore, 38 families were included in the whole study. Those recruited in June, September and November 2017; January, March and April 2018; and November 2019 were included in the rhythmic training (RTr+, N = 20). Among these, one family refused, two could not complete all required training sessions due to scheduling problems, and two could not complete the post-training assessment because of the suspension of all non-essential activities imposed by the Italian government in order to contain the spread of COVID-19. Families recruited outside of the mentioned time periods were included in the control group (RTr−, N = 18). Among these, three could not complete the post-training assessment due to scheduling problems and one completed the assessment once they were outside of the required age range. Therefore, the final sample with complete behavioral data consisted of 15 infants for the RTr+ group and 14 infants for the RTr− group (see the flow diagram of participants in Figure 1).

The two groups did not differ in terms of sex (χ^2^ (1, N = 29) = 0.829, *p* = 0.362) or sociodemographic characteristics (Table 1A).

Electrophysiological data were collected at pre-training and post-training only for the RTr+ children (see Section 2.3.3). Data from four children were rejected due to insufficient artifact-free trials/refusal to wear the net at one of the two time-points. Since the RTr− group underwent a different electrophysiological task, data from the 11 RTr+ children with good ERP data were analyzed and compared to a sample of 14 children whose ERP data were previously collected using the same task [6,14]. Information about musical activity or other training was collected for the ERP control group in order to assure that they had not participated in any activity during the 6–12 months age range. This group also did not differ from the RTr+ group in terms of sex (χ^2^ (1, N = 25) = 0.337, *p* = 0.561) or for any sociodemographic variable (Table 1B).

### 2.2. Procedure and Study Design

Pre-training assessment occurred when infants were aged 6 months, 15 days to 8 months, 15 days (RTr+: M = 7.40 months, SD = 0.47; RTr−: M = 7.71 months, SD = 0.47). At this time-point, sociodemographic information was collected using questionnaires, including the Brief Music Experience Questionnaire (BMEQ) [45] to quantify families’ experience of music (see Section 2.3.2). Language and cognitive skills of infants were assessed using the Bayley Scales of Infant and Toddler Development, Third Edition [46] (see Section 2.3.1). In addition, only RTr+ infants were tested with the oddball ERP task (see Section 2.3.3). After the pre-training assessment, only RTr+ infants and their caregivers attended six sessions of training (see Section 2.4). In order to evaluate the effectiveness of the rhythmic training, a post-training assessment was administered when infants were aged 12 months, 1 day to 14 months, 1 day (RTr+: M = 12.5 months, SD = 0.60; RTr−: M = 12.83 months, SD = 0.69). At the post-training assessment, children were assessed again using the Expressive and Receptive scales of the Bayley Scales of Infant and Toddler Development, Third Edition and the ERP task (only RTr+ infants).

### 2.3. Tools

#### 2.3.1. Bayley Scales of Infant and Toddler Development

The Bayley Scales of Infant and Toddler Development, Third Edition [46] are an individually administered test intended to assess the developmental functioning of infants and children between the ages of 1 and 42 months. They are composed of five scales: Cognitive, Language (including the Receptive and Expressive Communication subtests), Motor (including the Fine and Gross Motor subtests), Socio-emotional and Adaptive Behavior. The Cognitive, Language and Motor scales are directly administered to the child, and items are arranged in an increasing order of difficulty. Each item is scored 1 point if passed and 0 points if failed; the total raw score for each subtest can be turned into scaled scores (M = 10; SD = 3) on the basis of the age range. For the purpose of this study, the Cognitive scale was administered during the first visit to rule out any intellectual deficiency, and the Language scales were used as pre-training and post-training assessment. Specifically, in the age range of our interest, the Receptive subtest of the Language scale (Receptive Communication, RC) investigates the infants’ pre-verbal behavior and verbal comprehension. The Expressive subtest (Expressive Communication, EC) investigates pre-verbal communication such as babbling and gestures, and early speech. Since the two groups were matched for age both at pre-training and post-training, total raw scores for each subtest were used in the analysis.

#### 2.3.2. The Brief Music Experience Questionnaire

The Brief Music Experience Questionnaire (BMEQ) [45] is a 53-item self-report measure of individual differences in people’s experience of music. Each item is scored on a 5-point Likert scale from 1 (very untrue) to 5 (very true). The questionnaire investigates people’s thoughts about music, their feelings and reactions towards it, and how it relates to their activities. The scoring produces six scales: Commitment to music, Innovative musical aptitude, Social uplift, Affective reactions, Positive psychotropic effects, Reactive musical behavior.

#### 2.3.3. EEG paradigm, Data Acquisition and Pre-Processing

**Figure 2:**
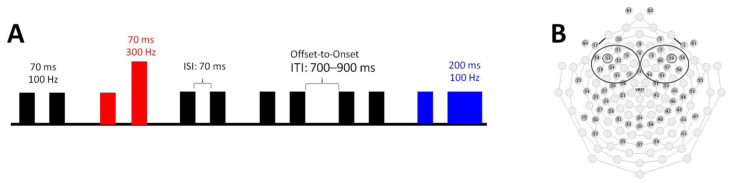
(**A**) Schematic representation of the multi-feature oddball paradigm; (**B**) selected electrode and cluster placement.

The electrophysiological functioning underlying auditory processing was explored by means of a non-speech multi-feature paradigm (extensively described previously [6,13,14,15,47]). In this paradigm, pairs of complex tones with an interstimulus interval (ISI) of 70 ms were presented. Standard tone pairs (STD stimuli) consisted of two identical 70 ms long 100 Hz tones, whereas in Deviant for Frequency (DEVF) stimuli, the second tone differed in fundamental frequency (300 Hz), and in Deviant for Duration (DEVD) stimuli, the second tone differed in duration (200 ms). The stimuli were presented in a passive oddball paradigm, in which 1200 stimuli (80% STD, 10% DEVF, 10% DEVD) were delivered in a pseudo-random order (at least three STD were presented before each DEV) (see Figure 2A; for a more complete description of the stimuli, see Cantiani et al. [6]).

During EEG recording (approximately 25 min), infants were seated on their caregiver’s lap in a sound-attenuated and electrically shielded room, and watched silent movies or were entertained with quiet toys. All stimuli were presented free field at an intensity of 75 dB. Auditory ERPs were recorded from 60 scalp sites using a dense-array EGI recording system (Electric Geodesic, Inc., Eugene, OR, USA) with Vertex as an online reference. The sampling rate was 250 Hz with an 0.1–100 Hz online bandpass filter.

After recording, the data were processed using EEGLAB [48] and ERPLAB [49], using procedures identical to those described in Cantiani et al. [6]. Pre-processing included offline bandpass filtering between 0.5 and 30 Hz, interpolation of noisy channels with a spherical spline (never >12 of the 60 channels) and re-referencing to the average. In order to ensure that EEG comparisons were as equitable as possible, only responses to the immediate pre-deviant STD were included in the average. The continuous EEG was segmented according to stimulus type (pre-deviant STD, DEVF and DEVD), with 100 ms pre-stimulus time (used for baseline correction) and 800 ms post-stimulus time. EEG epochs with artifacts were identified and rejected using both automatic criteria and visual inspection (for further information on ERP data processing, see Cantiani et al. [6]). A minimum of 60 artifact-free trials were used for averaging ERPs.

To examine the role of auditory processing, we focused on the components reflecting a neural change detection process, i.e., the large positive response corresponding to the mismatch response (MMR), the N2* peak (clearly identifiable only for DEVD), and the P2 peak (clearly identifiable only for DEVF at 12 months). Both the amplitude and latency of these peaks/components were analyzed. Time windows and electrode sites to be submitted to statistical analyses were selected based on previous studies [6,14]. For each participant, ERPs were extracted from a subset of 18 electrodes localized in the left and right frontocentral areas. Data were then averaged in two clusters corresponding to the left and right frontocentral areas, each including nine channels (Figure 2B; see Cantiani et al. [6] for details).

Peak (or mean) amplitude and peak latency were calculated for different time windows:

1. N2*: 250–400 ms for DEVD (peak amplitude and latency).
2. P2: 250–350 ms for DEVF (different waveform, peak amplitude and latency).
3. MMR: 350–550 ms for STD and DEVF and 420–620 ms for DEVD (different waveforms, mean amplitude).

### 2.4. Early Rhythmic Training

The experimental training consists of ecological and non-invasive training providing exposure to and active synchronization with complex musical rhythms. It was adapted from the BabyRhythms music program for infants implemented by Dr. Musacchia [50], based on the complex rhythms and traditional music of different cultures. This program is designed to promote the infant’s ability to recognize and process the complex rhythms of spoken language. Importantly, it is designed to tap into and empower basic auditory processing and is expected to modify the underlying neural mechanisms.

The training took place in small groups of infant–caregiver pairs (N = 3 to 5) for 1 h/week for 6 weeks. All training sessions were provided by two psychologist researchers who had been trained in the specific training protocol, including participation in a workshop led by Dr. Musacchia. According to recently implemented parent training [51], indications were given to caregivers to continue the training at home. At the end of each session, all music tracks were sent to parents. In addition, the parents were given a diary to be returned at the subsequent session. They were asked to fill it out daily, reporting how long they trained for and with which specific task.

The training includes several tasks described in the literature, for example, *tapping* and *bouncing* [52,53] at the beat of complex musical rhythms. Both these tasks are characterized by strong multi-sensory involvement (auditory, visual, and proprioceptive skills are simultaneously involved). The tapping task consisted of hand drumming (with alternating hand motions) on different sized drums (including experience of a “drum circle”). Bouncing to complex rhythms was provided using fitness balls, on which caregivers were asked to bounce together with the baby. Another task included in the training is called *dancing and freezing*, consisting of dancing at the tempo of the music and stopping any other auditory stimulation and movements when the musical stimulation stopped with unpredictable timing. Further tasks were related to *sound intensity*, consisting of playing small percussion instruments (maracas, egg shakers, rattles), following the tempo and, importantly, the intensity of music, and *multisensory stimulation*, consisting of stimulating the baby with colored fabrics or a playful parachute flapped in time with the tempo of the music.

The training sessions took place in a large, bright, quiet room. Each session was structured as follows. Firstly, infants were left free to explore the environment and the simple percussive musical toys for a few minutes. Then, a routine was established, providing an easy song that allows the participants to greet all infants personally and repeat their names. After this introduction, the activities started in the following order: tapping, bouncing, dancing and freezing, sound intensity (around five minutes for each task). After a short pause (five to ten minutes), the activities re-started in the following order: tapping, bouncing, dancing and freezing, multisensory stimulation (around five minutes for each task). Each session was concluded with a final easy “goodbye” song. The order of music tracks across different training groups was always kept identical, with the exception of the last training session, for which—in order to encourage caregiver engagement—we asked caregivers to choose the sequence across all the music tracks available.

Traditional music of different cultures was selected, including Western and Non-Western (e.g., Balkanic, African) music. They were selected to vary in metrics (including simple, e.g., 3/4, 4/4, compound, e.g., 6/8, and complex, e.g., 7/8 m), in tempo (“Andante” to “Allegro”; range, 76–156 beats per minute), and voices (for songs). All music tracks were played through two speakers at a comfortable listening level of around 70 decibels.

Parents and infants attended, on average, 83% of the training group sessions, i.e., about 5 out of 6 sessions. None of the participants missed the first group session, in which all tasks were carefully explained. When parents and infants missed one of the following group sessions, they were updated about the missed session’s contents and they received all of the music tracks to continue the training at home. On average, parents reported that over the training period, they performed at home with their infants 36 tasks (SD = 24.3, range 10–91), divided as follows: tapping, M = 11, SD = 6.9; bouncing, M = 7.8, SD = 6.1; dancing and freezing, M = 7.5, SD = 6.2; other, M = 9.8, SD = 8.6.

Finally, at the end of the last training session, parents were asked to express, anonymously, their satisfaction with the contents and methods of the training using five-point Likert scale questions (1 = very unsatisfied; 2 = unsatisfied; 3 = neutral; 4 = satisfied; 5 = very satisfied). The mean rating of parental responses revealed that parents were very satisfied or satisfied (M = 4.5, SD = 0.5).

### 2.5. Statistical Analyses

To compare the two groups prior to training, independent samples *t*-tests were performed to assess differences in (1) baseline language skills measured via the Bayley Scales; and (2) families’ general experience with music measured via the BMEQ. Welch’s *t*-test *t*- and *p*-values were reported when the assumption of equal variances was violated. To evaluate the effectiveness of the training on linguistic outcomes, two 2 × 2 repeated-measure ANOVAs were performed for each Bayley Language subtest, including the between-subject factor Group (RTr− vs. RTr+) and the within-subject factor Time (pre-training vs. post-training).

A group comparison was also made for the electrophysiological functioning underlying auditory processing. Independent sample *t*-tests were performed for amplitude and latency on each pre-training component to investigate baseline group differences in the ERP waveforms. Finally, as a preliminary assessment of the effect of training, repeated-measures ANOVAs were performed on the different peaks/components of interest. Specifically, a 2 × 2 × 2 ANOVA was performed for the N2* peak amplitude and latency for DEVD, including the between-subject factor Group (RTr− vs. RTr+) and the within-subject factors Hemisphere (Left vs. Right) and Time (pre-training vs. post-training); a 2 × 2 ANOVA was performed for the P2 peak amplitude and latency for DEVF on the difference waveform (Group × Hemisphere); a 2 × 2 × 2 × 2 ANOVA was performed for mean amplitude of the MMR on the difference waveform, including the between-subject factor Group (RTr− vs. RTr+) and the within-subject factors Hemisphere (Left vs. Right), Time (pre-training vs. post-training), and Condition (DEVF vs. DEVD).

## 3. Results

### 3.1. Behavioral Pre-Training Assessment

Measures collected during the pre-training assessment were compared via independent sample *t*-tests in order to exclude any group differences. The two groups did not significantly differ in their baseline language skills, expressed as total raw scores of Receptive and Expressive subtests of the Bayley Scales (see Table 2A,B). No differences between the two groups were found when comparing the parents’ experience of music (*p* > 0.05); descriptive statistics and group comparisons for every BMEQ scale are reported in Table S1.

### 3.2. Effect of Training on Language Skills

**Figure 3:**
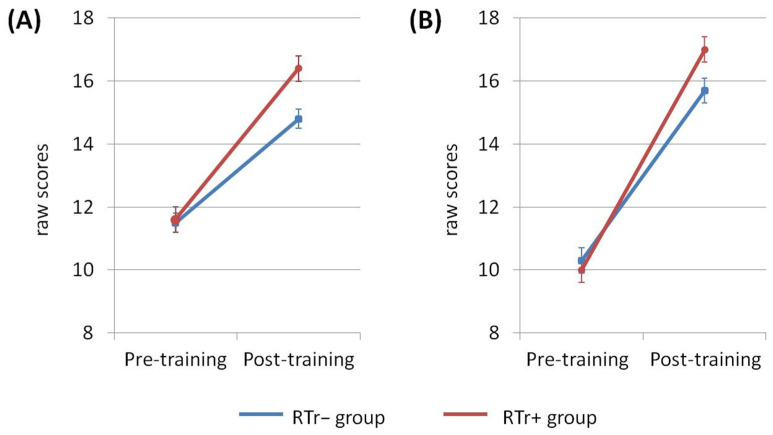
Descriptive graphical representation of the pre-training vs. post-training comparison for (**A**) the Bayley Receptive Communication and (**B**) Expressive Communication subtests. Error bars indicate SEM.

The repeated-measures ANOVA for the Bayley Receptive Communication subtest revealed a significant Group × Time interaction (F(1,27) = 4.326, *p* = 0.047, η^2^ = 0.138): the total raw score was higher at post-training for the RTr+ group (see Figure 3A, Table 2A). Likewise, the ANOVA performed on the Expressive Communication subtest revealed a significant Group × Time interaction (F(1,27) = 4.600, *p* = 0.041, η^2^ = 0.146), in which the RTr+ group showed a larger increase in the total raw score between pre-training and post-training compared to the RTr− group (see Figure 3B, Table 2B).

### 3.3. Preliminary Findings on the ERP Oddball Paradigm

**Figure 4:**
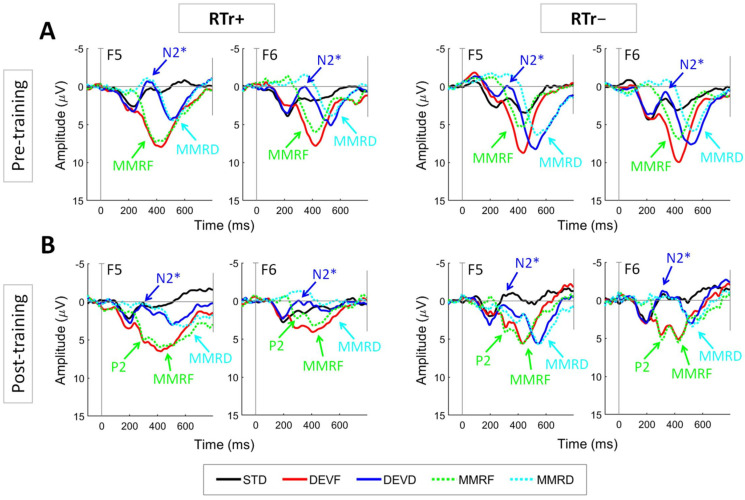
Grand average waveforms for RTr+ and RTr− infants at (**A**) pre-training and (**B**) post-training. Representative channels F5 and F6 located, respectively in the left and right frontocentral regions are shown. The standard waveform (STD, black line) is plotted against the waveforms for the frequency deviant (DEVF, red line) and the duration deviant (DEVD, blue line). In addition, the difference waveforms relative to MMRF (DEVF minus STD; green dotted line) and MMRD (DEVD minus STD; light-blue dotted line) are plotted. Plots were filtered using a 20 Hz low-pass filter for presentation purposes only. Negative voltage is plotted upward.

First, analyses of the ERP pre-training data were conducted to ensure that there were no systematic initial group differences. Figure 4A shows the pre-training grand average waveforms for the RTr+ and RTr− groups. As expected, independent *t*-tests comparing the two groups at pre-training revealed no significant differences in any of the indices considered in this study (*p* > 0.05; Table S2). Figure 4B shows the post-training grand average waveforms for the RTr+ and RTr− groups. As expected from the previous literature, a qualitative analyses of the morphology of the waveforms revealed age-related changes, including decreased amplitude of the MMR, and the emergence of additional peaks (i.e., the P2 peak; [5,17]).

**Figure 5:**
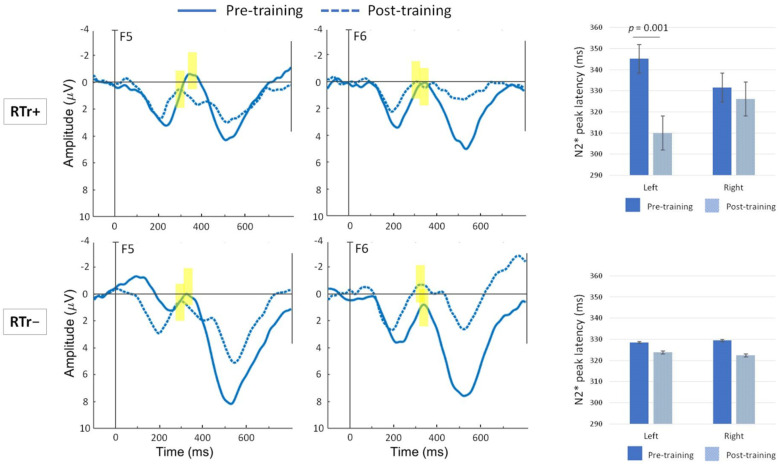
Grand average waveforms are shown for the DEVD condition, where the N2* peak is maximal. The pre-training waveform (solid line) is plotted against the post-training waveform (dotted line) for RTr+ and RTr− infants. Representative channels F5 and F6 located, respectively, in the left and right frontocentral regions are shown. Highlighted yellow bars indicate the location of the maximum N2* peak. Plots were filtered using a 20 Hz low-pass filter for presentation purposes only. Negative voltage is plotted upward. On the right, bar graphs (error bars indicate SEM) show the mean N2* peak latency.

Furthermore, for preliminary assessment of the effect of the training, repeated-measures ANOVAs were performed on the different peaks/components of interest. 2 (Group) × 2 (Hemisphere) × 2 (Time) ANOVAs were run on amplitude and latency of the N2* peak in the DEVD condition. A significant three-way interaction emerged for latency [F (1, 23) = 5.145, *p* = 0.033, η^2^ = 0.183]. Although the latency of the N2* peak was overall faster at the post-training recording (M = 320.9, SD = 18.9) than at pre-training (M = 333.1, SD = 17.2; *p* = 0.011), the age-dependent shift of latency was higher in the left hemisphere for the RTr+ group (*p* = 0.001, see Figure 5).

**Figure 6:**
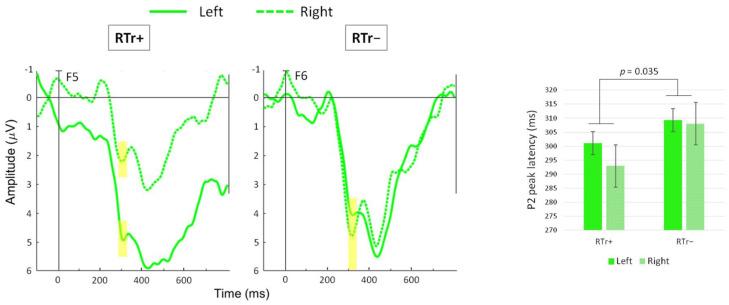
Grand average difference waveforms (DEVF minus STD) are shown, where the P2 peak is maximal. The waveform from channel F5 (representative of the left frontocentral region, solid line) is plotted against the waveform from channel F6 (representative of the right frontocentral region, dotted line) for RTr+ and RTr− infants. Highlighted yellow bars indicate the location of the maximum P2 peak. Plots were filtered using a 20 Hz low-pass filter for presentation purposes only. Negative voltage is plotted upward. On the right, bar graphs (error bars indicate SEM) show the mean P2 peak latency.

2 (Group) × 2 (Hemisphere) ANOVAs were run on amplitude and latency of the post-training P2 peak for DEVF on the difference waveform. A significant main effect of Group emerged for latency [F(1,23) = 4.999, *p* = 0.035, η^2^ = 179]. At age 12 months, the latency of the P2 peak was overall faster for the RTr+ group (M = 297.0, SD = 15.0) than the RTr− group (M = 308.6, SD = 11.1; see Figure 6).

No significant amplitude differences were observed for the N2* and P2 peaks. Similarly, no significant differences between groups were found concerning mean amplitude of the MMR, explored using a 2 (Group) × 2 (Time) × 2 (Hemisphere) × 2 (Condition) ANOVA. Concerning this last analysis, only a main effect of Time (F(1,23) = 5.529, *p* = 0.028, η^2^ = 0.194) emerged, confirming the expected age-related decrease in MMR amplitude, which was similar in the RTr+ and RTr− groups.

## 4. Discussion

The present study aimed at investigating the feasibility and efficacy of early rhythmic training on typically developing infants. Firstly, we focused on the impact of training on early linguistic outcomes; moreover, we preliminarily investigated the effects on the electrophysiological components that reflect auditory processing.

Results on the linguistic outcome show that the infants who attended the training displayed a significantly larger improvement than the control group, both in terms of receptive and expressive function. At the electrophysiological level, the expected age-related changes from pre-training to post-training were observed, with some group differences. Specifically, the N2* peak latency for the duration deviant in the left hemisphere decreased significantly more in the RTr+ group; the P2 peak latency for the frequency deviant (difference waveform) was faster overall for this group as well.

Considering language acquisition at the behavioral level, our results seem promising and agree with other studies of older populations. Previous findings on typically developing children [26,27,54], as well as on children with LD or DLD [30,55], have established the beneficial role of musical expertise and training on different aspects of the language domain, in line with the hypothesis of the transfer of skills from music to language [56]. Furthermore, newborns and infants in the first year of life can perceive auditory aspects of language that share many features with music [57]; given the maximal neural plasticity at this age, the positive effects on language abilities of an enriched active auditory and musical experience can be expected to be seen, even at this early stage. However, little is currently known about the effects of musical training on such early linguistic outcomes. Gerry and colleagues [40] showed how an active musical experience between 6 and 12 months of age can facilitate the use of earlier prelinguistic communicative gestures, as opposed to merely passive exposure to recorded music. Similarly, the training presented here seems to boost the typical trajectory of language development, as shown by the higher increase of the RTr+ group in the Bayley raw scores, corresponding to a wider and more complex repertoire of both receptive and expressive prelinguistic and linguistic skills. Taken together, these results seem to suggest that early active musical training can, in fact, enhance language development even at its very first stages (i.e., pre-verbal and verbal comprehension, communicative behavior and gestures, babbling and first words). Interestingly, when converting raw scores into scaled scores, it can be observed that 67% of our RTr+ infants display an improvement of two points from pre-training to post-training in at least one subtest, with two infants scoring more than one standard deviation above average and one infant scoring more than two. In the RTr− group, significantly fewer infants show a two-points increase (only 29%), and all of their scores place within the average range. Unfortunately, it has not been possible to keep further track of the development of most of the children involved in this study due to the COVID-19-related suspension of activities; therefore, these findings should be interpreted cautiously. Even though the positive effects seen post-training seem encouraging, a longer follow-up would be required to assess what quantity and quality of this early training is enough for the children to maintain the advantage they gained, even at later and more complex stages of language development.

Linguistic skills are known to be strongly based on the general-domain ability of auditory processing, and the way this ability develops over time has been investigated. A few studies have assessed the maturational profiles of EEG/ERP in response to auditory stimuli in typically developing infants [5,58,59]. Overall, the results show that, with age, the latency of the peaks decreases (i.e., the responses tended to peak earlier in time [5,59]) and the amplitude of the large mismatch-like response decreases (i.e., mean amplitude of the MMR tended to be less positive [5]). Moreover, the appearance of new peaks over age is also described; for example, Choudhury and Benasich [5] observed the emergence of a positive deflection (P2) beginning at about 12 months.

Here, we described for the first time the age-related maturational effects between 6–8 and 12–14 months of age. Consistent with the previous findings, at age 12–14 months, we observed decreased latency of the N2* peak (DEVD condition), amplitude reduction in the MMR (DEV–STD difference waveforms) and the emergence of a clear P2 peak (especially for the DEVF condition, DEVF–STD difference waveform).

Interestingly, as preliminary findings suggest the efficacy of our early rhythmic intervention on auditory ERP, we found group effects on peak latency that go beyond maturation and that might reflect an enhancement in the speed of acoustic processing as a result of attending the rhythmic training. Specifically, the latency of the change discrimination N2* peak decreased by an average of 12 ms over 6 months, but in the group of children who attended the training (RTr+), we observed a latency decrease of over 30 ms in the left frontocentral region. Group differences also emerged for the latency of the P2 in the post-training assessment, with the RTr+ group achieving significantly faster latencies (around 12 ms) for the P2 peak of the DEVF–STD difference waveform. These results are in line with previous findings, also reporting faster P2 and N2* peaks reflecting the auditory processing efficiency enhanced by early interactive acoustic experience [17]. Since previous studies suggested that latency reduction could be associated with myelination and synaptic efficiency [5], we might speculate that it is at this level that our rhythmic training may exert its effect. The fact that this improvement in the N2* peak is evident in the left hemisphere is not surprising, since in typical development, we expect an early left hemisphere specialization for temporal processing [60,61] and—more specifically—for the processing of rapidly presented non-speech auditory stimuli [13]. Interestingly, recent follow-up analyses of Benasich et al.’s study [17] hypothesized that the reported changes might be supported by more mature left-lateralized patterns of oscillatory activity for tones [18] and speech [19]. More sophisticated analytic strategies that allow age-appropriate source localization and fine-grained time frequency analyses [13,62,63] could be applied to this dataset as well, in order to gain more information about the involved neural sources and mechanisms. It is worth noting that in the present study, no changes attributable to the training occurred for the amplitude of the MMR, reflecting the neural process associated with change detection [58]. These preliminary results point to the direction that attending the rhythmic training had specific neural effects on the speed of acoustic processing.

These results, although preliminary, expand our limited knowledge of the efficacy of early music training in improving infants’ neural processing of auditory stimuli. In particular, our results build on the findings by Zhao and Kuhl [41], who explored the effect of music training on infants’ neural processing using magnetoencephalography. In this study, the group of infants who attended the music intervention exhibited significantly larger MMRs in response to violations of music temporal structure, but also of speech temporal structure, suggesting generalization.

Some limitations of the present study should be mentioned. First, the limited sample size, especially for the subsamples with ERP data (N = 11 vs. 14), does not allow us to draw firm conclusions; further studies including larger samples are needed. Unfortunately, the current project has been suspended since March 2020 because of the limitations imposed to contain the spread of COVID-19: the importance of the social context in facilitating movement synchronization during joint rhythmic activity [64] prevented us from implementing individualized or remote versions of the training. A second limitation of the study is the shortness of the longitudinal investigation and the absence of further follow-up to delineate the effects of the training on later development. Most of the in-person evaluations were scheduled at later ages, again due to the COVID-19 lockdowns imposed by the Italian Government, resulting in an unfortunate loss of this important information. Further studies should fill this gap. Finally, the lack of a control training program is a further limitation of the present study. Here, we have compared the group of children who participated in the training with two different groups of children who did not attend specific activities in the considered time frame. Ideally, the study’s conclusions would benefit from the presence of a group of children who attended a different kind of training (e.g., different social play sessions not involving auditory and rhythmic stimulation, as in previous studies [41]). The presence of such a group could help rule out possible effects of child and/or parent engagement and check for the specificity of the effects. Adding further complexity, the optimal control training could include the same auditory and rhythmic stimulation as in the experimental training, but without requiring active infant participation. Based on previous evidence, we expect that only active participation—and not passive listening—would enhance language skills and their electrophysiological underpinnings [17,40,42].

Alongside these limitations, the strengths of our study should be acknowledged. The main strength of the study is that we were successful in demonstrating the feasibility of such an early, active and ecological training. The training was welcomed by the parents, who positively rated their satisfaction with the contents and methods and were willing to continue the activities at home. Moreover, the RTr+ and control groups were well characterized and matched at pre-training for sociodemographic and environmental variables (including families’ music experience).

## 5. Conclusions

Our results show preliminary evidence on the feasibility and efficacy of an early rhythmic training on typically developing infants’ auditory and language abilities.

Next steps include testing the efficacy of the training on a sample of infants at high familial risk for DLD and LD, who are expected to show early impairment in auditory processing skills [5,6,11,12] and, thus, might benefit from the training even more than not-at-risk infants, in terms of improvements in both acoustic and linguistic skills. In this sense, we may be able to modify their atypical developmental trajectories before the emergence and crystallization of any symptoms in the language domain.

## Figures and Tables

**Table 1:** Descriptive statistics: Mean (standard deviation) and group comparison of sociodemographic characteristics.

**A**				
	**RTr+** **(N = 15)**	**RTr−** **(N = 14)**	**t (df)**	***p***
Socioeconomic status ^a^	68.00 (16.56)	70.00 (17.97)	0.312 (27)	0.758
Maternal education level ^b^	63.33 (15.89)	64.29 (10.16)	0.191 (27)	0.850
Paternal education level ^b^	55.00 (13.76)	50.71 (19.79)	−0.681 (27)	0.502
**B**				
	**RTr+** **Subsample with ERP Data** **(N = 11)**	**RTr−** **Sample with ERP Data** **(N = 14)**	**t (df)**	***p***
Socioeconomic status ^a^	58.18 (17.22)	66.07 (12.43)	0.164 (23)	0.196
Maternal education level ^b^	59.09 (18.14)	59.29 (13.28)	0.031 (23)	0.976
Paternal education level ^b^	51.36 (13.43)	55.38 (8.77)	0.881 (23)	0.388

^a^ Nine-point scale, whereby a score ranging from 10 to 90 was assigned to each parental job and the higher of the two scores was used when both parents were employed [44]. ^b^ Nine-point ordinal scale, created ad hoc and based on the Italian school system.

**Table 2:** Mean (standard deviation) of the Bayley Language scale raw scores and Group comparisons at pre- and post-training.

**(A) Bayley Language Scale—Receptive Communication Subtest**				
	**RTr−**	**RTr+**	***t* (27)**	***p***
Pre-training	11.46 (1.34)	11.60 (0.74)	−0.335	0.735
Post-training	14.86 (1.23)	16.40 (2.23)	−2.283	0.031
**(B) Bayley Language Scale—Expressive Communication Subtest**				
	**RTr−**	**RTr+**	***t* (27)**	***p***
Pre-training	10.32 (1.64)	10.00 (1.54)	0.546	0.590
Post-training	15.79 (1.63)	17.00 (1.93)	−1.827	0.079

## Data Availability

The data that support the findings of this study are available from the corresponding author, C.C., upon reasonable request.

## Supplementary Materials

The following are available online at https://www.mdpi.com/article/10.3390/brainsci11111546/s1, Table S1: Mean (Standard Deviation) and group comparison for parents’ BMEQ scales, Table S2: Mean (Standard Deviation) and group comparison for pre-training ERP indices considered in the study.

## References

[1] Endress A.D., Nespor M., Mehler J. (2009). Perceptual and memory constraints on language acquisition. Trends Cogn. Sci..

[2] Saffran J.R., Estes K.G. (2006). Mapping sound to meaning: Connections between learning about sounds and learning about words. Adv. Child Dev. Behav..

[3] Hannon E.E., Trainor L.J. (2007). Music acquisition: Effects of enculturation and formal training on development. Trends Cogn. Sci..

[4] Benasich A.A., Tallal P. (2002). Infant discrimination of rapid auditory cues predicts later language impairment. Behav. Brain Res..

[5] Choudhury N., Benasich A.A. (2011). Maturation of auditory evoked potentials from 6 to 48 months: Prediction to 3 and 4 year language and cognitive abilities. Clin. Neurophysiol..

[6] Cantiani C., Riva V., Piazza C., Bettoni R., Molteni M., Choudhury N., Marino C., Benasich A.A. (2016). Auditory discrimination predicts linguistic outcome in Italian infants with and without familial risk for language learning impairment. Dev. Cogn. Neurosci..

[7] Lohvansuu K., Hämäläinen J.A., Ervast L., Lyytinen H., Leppänen P.H.T. (2018). Longitudinal interactions between brain and cognitive measures on reading development from 6 months to 14 years. Neuropsychologia.

[8] Van Zuijen T.L., Plakas A., Maassen B.A.M., Been P., Maurits N.M., Krikhaar E., van Driel J., van der Leij A. (2012). Temporal auditory processing at 17 months of age is associated with preliterate language comprehension and later word reading fluency: An ERP study. Neurosci. Lett..

[9] Hämäläinen J.A., Salminen H.K., Leppänen P.H.T. (2012). Basic Auditory Processing Deficits in Dyslexia: Systematic Review of the Behavioral and Event-Related Potential/ Field Evidence. J. Learn. Disabil..

[10] Cantiani C., Lorusso M.L., Valnegri C., Molteni M. (2010). Perception of non-verbal auditory stimuli in Italian dyslexic children. Dev. Neuropsychol..

[11] Choudhury N., Leppanen P.H.T., Leevers H.J., Benasich A.A. (2007). Infant information processing and family history of specific language impairment: Converging evidence for RAP deficits from two paradigms. Dev. Sci..

[12] Leppänen P.H.T., Richardson U., Pihko E., Eklund K.M., Guttorm T.K., Aro M., Lyytinen H. (2010). Brain Responses to Changes in Speech Sound Durations Differ Between Infants With and Without Familial Risk for Dyslexia. Dev. Neuropsychol..

[13] Cantiani C., Ortiz-Mantilla S., Riva V., Piazza C., Bettoni R., Musacchia G., Molteni M., Marino C., Benasich A.A. (2019). Clinical Reduced left-lateralized pattern of event-related EEG oscillations in infants at familial risk for language and learning impairment. Neuroimage Clin..

[14] Riva V., Cantiani C., Mornati G., Gallo M., Villa L., Mani E., Saviozzi I., Marino C., Molteni M. (2018). Distinct ERP profiles for auditory processing in infants at-risk for autism and language impairment. Sci. Rep..

[15] Piazza C., Cantiani C., Akalin-Acar Z., Miyakoshi M., Benasich A.A., Reni G., Bianchi A.M., Makeig S. (2016). ICA-derived cortical responses indexing rapid multi-feature auditory processing in six-month-old infants. Neuroimage.

[16] Riva V., Cantiani C., Benasich A.A., Molteni M., Piazza C., Giorda R., Dionne G., Marino C. (2018). From CNTNAP2 to Early Expressive Language in Infancy: The Mediation Role of Rapid Auditory Processing. Cereb. Cortex.

[17] Benasich A.A., Choudhury N.A., Realpe-Bonilla T., Roesler C.P. (2014). Plasticity in Developing Brain: Active Auditory Exposure Impacts Prelinguistic Acoustic Mapping. J. Neurosci..

[18] Musacchia G., Ortiz-Mantilla S., Choudhury N., Realpe-Bonilla T., Roesler C., Benasich A.A. (2017). Active auditory experience in infancy promotes brain plasticity in Theta and Gamma oscillations. Dev. Cogn. Neurosci..

[19] Ortiz-Mantilla S., Realpe-Bonilla T., Benasich A.A. (2019). Early Interactive Acoustic Experience with Non-speech Generalizes to Speech and Confers a Syllabic Processing Advantage at 9 Months. Cereb. Cortex.

[20] Kraus N., Skoe E., Parbery-Clark A., Ashley R. (2009). Experience-induced Malleability in Neural Encoding of Pitch, Timbre, and Timing: Implications for Language and Music. Ann. N. Y. Acad. Sci..

[21] Patel A.D. (2014). Can nonlinguistic musical training change the way the brain processes speech? The expanded OPERA hypothesis. Hear. Res..

[22] Kraus N., Chandrasekaran B. (2010). Music training for the development of auditory skills. Nat. Rev. Neurosci..

[23] Pantev C., Oostenveld R., Engelien A., Ross B., Roberts L.E., Hoke M. (1998). Increased auditory cortical representation in musicians. Nature.

[24] Parbery-Clark A., Tierney A., Strait D.L., Kraus N. (2012). Musicians have fine-tuned neural distinction of speech syllables. Neuroscience.

[25] Zuk J., Ozernov-Palchik O., Kim H., Lakshminarayanan K., Gabrieli J.D.E., Tallal P., Gaab N. (2013). Enhanced Syllable Discrimination Thresholds in Musicians. PLoS ONE.

[26] Moreno S., Marques C., Santos A., Santos M., Castro S.L., Besson M. (2009). Musical Training Influences Linguistic Abilities in 8-Year-Old Children: More Evidence for Brain Plasticity. Cereb. Cortex.

[27] Moreno S., Bialystok E., Barac R., Schellenberg E.G., Cepeda N.J., Chau T. (2011). Short-Term Music Training Enhances Verbal Intelligence and Executive Function. Psychol. Sci..

[28] Bhide A., Power A., Goswami U. (2013). A Rhythmic Musical Intervention for Poor Readers: A Comparison of Efficacy With a Letter-Based Intervention. Mind Brain Educ..

[29] Kraus N., Slater J., Thompson E.C., Hornickel J., Strait D.L., Nicol T., White-Schwoch T. (2014). Music Enrichment Programs Improve the Neural Encoding of Speech in At-Risk Children. J. Neurosci..

[30] Flaugnacco E., Lopez L., Terribili C., Montico M., Zoia S., Schön D. (2015). Music Training Increases Phonological Awareness and Reading Skills in Developmental Dyslexia: A Randomized Control Trial. PLoS ONE.

[31] Musacchia G., Strait D., Kraus N. (2008). Relationships between behavior, brainstem and cortical encoding of seen and heard speech in musicians and non-musicians. Hear. Res..

[32] Wong P.C.M., Skoe E., Russo N.M., Dees T., Kraus N. (2007). Musical experience shapes human brainstem encoding of linguistic pitch patterns. Nat. Neurosci..

[33] Doelling K.B., Poeppel D. (2015). Cortical entrainment to music and its modulation by expertise. Proc. Natl. Acad. Sci. USA.

[34] Herholz S.C., Zatorre R.J. (2012). Musical Training as a Framework for Brain Plasticity: Behavior, Function, and Structure. Neuron.

[35] Gaser C., Schlaug G. (2003). Gray Matter Differences between Musicians and Nonmusicians. Ann. N. Y. Acad. Sci..

[36] Ohnishi T., Matsuda H., Asada T., Aruga M., Hirakata M., Nishikawa M., Katoh A., Imabayashi E. (2001). Functional Anatomy of Musical Perception in Musicians. Cereb. Cortex.

[37] Schlaug G., Jäncke L., Huang Y., Staiger J.F., Steinmetz H. (1995). Increased corpus callosum size in musicians. Neuropsychologia.

[38] Winkler I., Háden G.P., Ladinig O., Sziller I., Honing H. (2009). Newborn infants detect the beat in music. Proc. Natl. Acad. Sci. USA.

[39] Gerry D.W., Faux A.L., Trainor L.J. (2010). Effects of Kindermusik training on infants’ rhythmic enculturation. Dev. Sci..

[40] Gerry D., Unrau A., Trainor L.J. (2012). Active music classes in infancy enhance musical, communicative and social development. Dev. Sci..

[41] Zhao T.C., Kuhl P.K. (2016). Musical intervention enhances infants’ neural processing of temporal structure in music and speech. Proc. Natl. Acad. Sci. USA.

[42] Virtala P., Partanen E. (2018). Can very early music interventions promote at-risk infants’ development?. Ann. N. Y. Acad. Sci..

[43] Partanen E., Kujala T., Tervaniemi M., Huotilainen M. (2013). Prenatal Music Exposure Induces Long-Term Neural Effects. PLoS ONE.

[44] Hollingshead A.B. Four Factor Index of Social Status; Yale University 1975. https://sociology.yale.edu/sites/default/files/files/yjs_fall_2011.pdf#page=21.

[45] Werner P.D., Swope A.J., Heide F.J. (2006). The Music Experience Questionnaire: Development and correlates. J. Psychol..

[46] Bayley N. (2006). Bayley Scales of Infant and Toddler Development.

[47] Piazza C., Cantiani C., Miyakoshi M., Riva V., Molteni M., Reni G., Makeig S. (2020). EEG Effective Source Projections Are More Bilaterally Symmetric in Infants Than in Adults. Front. Hum. Neurosci..

[48] Delorme A., Makeig S. (2004). EEGLAB: An open source toolbox for analysis of single-trial EEG dynamics including independent component analysis. J. Neurosci. Methods.

[49] Lopez-Calderon J., Luck S.J., Heekeren H.R. (2014). ERPLAB: An open-source toolbox for the analysis of event-related potentials. Front. Hum. Neurosci..

[50] AbyRhythms—Music for Early Development. http://www.babyrhythms.com/.

[51] Weitzman E., Girolametto L., Drake L., McCauley R.J., Fey M.E., Gillam R.B. (2017). Hanen Programs® for parents: Parent implemented early language intervention. Treatment of Language Disorders in Children.

[52] Phillips-Silver J., Trainor L.J. (2005). Feeling the Beat: Movement Influences Infant Rhythm Perception. Science.

[53] Cirelli L.K., Einarson K.M., Trainor L.J. (2014). Interpersonal synchrony increases prosocial behavior in infants. Dev. Sci..

[54] Corrigall K.A., Trainor L.J. (2011). Associations Between Length of Music Training and Reading Skills in Children. Music Percept..

[55] Roden I., Früchtenicht K., Kreutz G., Linderkamp F., Grube D. (2019). Auditory Stimulation Training With Technically Manipulated Musical Material in Preschool Children With Specific Language Impairments: An Explorative Study. Front. Psychol..

[56] White E.J., Hutka S.A., Williams L.J., Moreno S. (2013). Learning, neural plasticity and sensitive periods: Implications for language acquisition, music training and transfer across the lifespan. Front. Syst. Neurosci..

[57] Brandt A.K., Slevc R., Gebrian M. (2012). Music and Early Language Acquisition. Front. Psychol..

[58] Kushnerenko E., Ceponiene R., Balan P., Fellman V., Huotilaine M., Näätäne R. (2002). Maturation of the auditory event-related potentials during the first year of life. Neuroreport.

[59] Morr M.L., Shafer V.L., Kreuzer J.A., Kurtzberg D. (2002). Maturation of mismatch negativity in typically developing infants and preschool children. Ear Hear..

[60] Dehaene-Lambertz G. (2017). The human infant brain: A neural architecture able to learn language. Psychon. Bull. Rev..

[61] Minagawa-Kawai Y., Cristià A., Dupoux E. (2011). Cerebral lateralization and early speech acquisition: A developmental scenario. Dev. Cogn. Neurosci..

[62] Musacchia G., Choudhury N.A., Ortiz-Mantilla S., Realpe-Bonilla T., Roesler C.P., Benasich A.A. (2013). Oscillatory support for rapid frequency change processing in infants. Neuropsychologia.

[63] Musacchia G., Ortiz-Mantilla S., Realpe-Bonilla T., Roesler C.P., Benasich A.A. (2015). Infant Auditory Processing and Event-related Brain Oscillations. J. Vis. Exp..

[64] Kirschner S., Tomasello M. (2009). Joint drumming: Social context facilitates synchronization in preschool children. J. Exp. Child Psychol..

