# Supplementary material for: Impact of Early Rhythmic Training on Language Acquisition and Electrophysiological Functioning Underlying Auditory Processing: Feasibility and Preliminary Findings in Typically Developing Infants
Source: Brain Sci. 2021 Nov 21;11(11):1546. doi: 10.3390/brainsci11111546 (PMC8615969; doi:10.3390/brainsci11111546)
Supplement: Supplementary file 1 [file brainsci-11-01546-s001.zip › brainsci-1446733-supplementary.pdf]

Table S1. Mean (Standard Deviation) and group comparison for parents' BMEQ scales.

Mothers' BMEQ scores				
	**RTr+**	**RTr–**	**t**	**p**
**Commitment to music**	1.96 (0.67)	2.03 (0.39)	0.267	0.792
**Innovative musical aptitude**	1.83 (0.80)	1.78 (0.90)	-0.153	0.880
**Social uplift**	3.50 (0.65)	3.16 (0.85)	-1.085	0.290
**Affective reactions**	4.09 (0.39)	4.11 (0.49)	0.095	0.925
**Positive psychotropic effects**	3.03 (0.59)	2.98 (0.52)	-0.233	0.818
**Reactive musical behavior**	3.69 (0.68)	3.57 (0.82)	-0.408	0.687
**Fathers' BMEQ scores**				
	**RTr+**	**RTr–**	**t**	**p**
**Commitment to music**	2.45 (0.73)	2.09 (0.58)	-1.310	0.204
**Innovative musical aptitude**	2.09 (0.63)	2.32 (1.02)	0.654	0.520
**Social uplift**	2.83 (0.69)	2.73 (0.59)	-0.394	0.697
**Affective reactions**	4.04 (0.39)	4.18 (0.37)	0.879	0.389
**Positive psychotropic effects**	3.11 (0.56)	3.01 (0.73)	-0.365	0.719
**Reactive musical behavior**	3.40 (0.58)	3.49 (0.84)	0.324	0.749

Table S2. Mean (Standard Deviation) and group comparison for pre-training ERP indices considered in the study.

	RTr+ subsample with ERP data (N = 11)	RTr– sample with ERP data (N = 14)	t	p
**N2* DEVD Left Amplitude**	-1.20 (1.03)	-1.18 (1.94)	0.020	0.985
**N2* DEVD Right Amplitude**	-0.83 (2.04)	-0.38 (2.83)	0.448	0.658
**N2* DEVD Left Latency**	345.21 (19.73)	328.48 (21.03)	-2.029	0.054
**N2* DEVD Right Latency**	331.60 (26.21)	329.37 (19.76)	-0.243	0.810
**MMR DEVF Left Mean Amplitude**	5.23 (2.59)	3.02 (2.75)	-2.045	0.052
**MMR DEVF Right Mean Amplitude**	3.46 (3.81)	3.94 (2.73)	0.365	0.718
**MMR DEVD Left Mean Amplitude**	2.87 (2.50)	3.51 (2.50)	0.638	0.530
**MMR DVD Right Mean Amplitude**	1.95 (2.01)	3.10 (3.18)	1.044	0.307
